# *Coriandrum sativum* L., essential oil as a promising source of bioactive compounds with GC/MS, antioxidant, antimicrobial activities*: in vitro* and *in silico* predictions

**DOI:** 10.3389/fchem.2024.1369745

**Published:** 2024-06-20

**Authors:** Ghizlane Nouioura, Mohamed El fadili, Naoufal El Hachlafi, Souad Maache, Ibrahim Mssillou, Hatem A. Abuelizz, Fatima Zahra Lafdil, Sara Er-rahmani, Badiaa Lyoussi, Elhoussine Derwich

**Affiliations:** ^1^ Laboratory of Natural Substances, Pharmacology, Environment, Modeling, Health and Quality of Life (SNAMOPEQ), Faculty of Sciences Dhar El Mehraz, Sidi Mohamed Ben Abdellah University, Fez, Morocco; ^2^ LIMAS Laboratory, Faculty of Sciences Dhar El Mehraz, Sidi Mohamed Ben Abdellah University, Fez, Morocco; ^3^ Laboratory of Microbial Biotechnology and Bioactive Molecules, Sciences and Technologies Faculty, Sidi Mohamed Ben Abdellah University, Fez, Morocco; ^4^ Department of Pharmaceutical Chemistry, College of Pharmacy, King Saud University, Riyadh, Saudi Arabia; ^5^ Laboratory of Bio-resources, Biotechnology, Faculty of Sciences, Ethnopharmacology and Health, Mohammed the First University, Oujda, Morocco; ^6^ Department of Chemistry, University of Torino, Torino, Italy; ^7^ Unity of GC/MS and GC, City of Innovation, Sidi Mohamed Ben Abdellah University, Fez, Morocco

**Keywords:** *Coriandrum sativum*, essential oil, antioxidant, antimicrobial, *in silico* predictions, GC-MS

## Abstract

**Introduction:** *Coriandrum sativum* L. essential oil (CS-EO) is being evaluated *in vitro* for its antioxidant and antimicrobial properties, and its volatile compounds are to be identified as part of this exploratory study.

**Methods:** The processes underlying the *in vitro* biological properties were explained using *in silico* simulations, including drug-likeness prediction, molecular docking, and pharmacokinetics (absorption, distribution, metabolism, excretion, and toxicity—ADMET). Chemical screening of CS-EO was conducted using gas chromatography-mass spectrometry (GC-MS). Five *in vitro* complementary techniques were used to assess the antioxidant activity of CS-EO: reducing power (RP), 2,2-diphenyl-1-picrylhydrazyl (DPPH), 2,2′-azinobis (3-ethylbenzothiazoline-6-sulfonate) (ABTS) radical scavenging activity, β-Carotene bleaching test (BCBT), and phosphomolybdenum assay (TAC).

**Results:** According to GC-MS analysis, linalool (59.04%), γ-Terpinene (13.02%), and α-Pinene (6.83%) are the main constituents of CS-EO. Based on the *in vitro* antioxidant assay results, CS-EO has been found to have a superior antioxidant profile. Its estimated scavenging rates for ABTS^+^ are 0.51 ± 0.04 mg/mL, BCBT is 9.02 ± 0.01 mg/mL, and CS-EO is 1.52 ± 0.14 mg/mL. *C. sativum* demonstrated 6.13 ± 0.00 μg/mL for reducing power and 213.44 ± 0.45 mg AAE/mL for total antioxidant activity. The *in vitro* antimicrobial activity of CS-EO was assessed against five strains, including two gram-positive bacteria, two gram-negative bacteria, and one fungal strain (*Candida albicans*). Significant antibacterial and antifungal activities against all strains were found using the disc-diffusion assay, with zones of inhibition larger than 15 mm. The microdilution test highlighted the lowest MIC and MBC values with gram-positive bacteria, ranging from 0.0612 to 0.125% v/v for MIC and 0.125% v/v for MBC. The fungal strain’s MFC was 1.0% v/v and its MIC was measured at 0.5%. Based on the MBC/MIC and MFC/MIC ratios, CS-EO exhibits bactericidal and fungicidal activity. The ADMET study indicates that the primary CS-EO compounds are good candidates for the development of pharmaceutical drugs due to their favorable pharmacokinetic properties.

**Conclusion:** These results point to a potential application of this plant as a natural remedy and offer empirical backing for its traditional uses. It is a promising environmentally friendly preservative that can be used extensively in the food and agricultural industries to prevent aflatoxin contamination and fungal growth in stored goods.

## Graphical Abstract

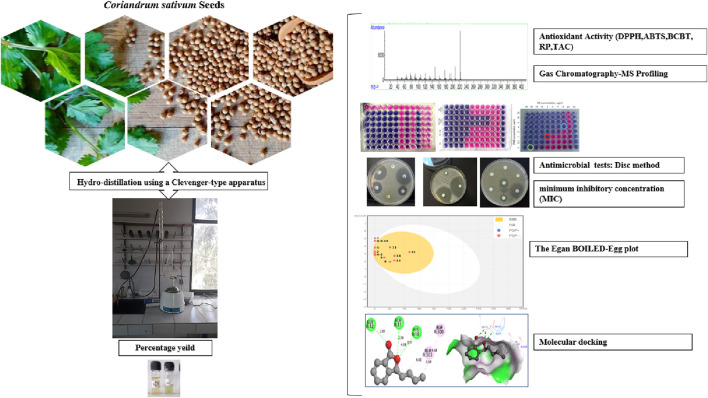

## 1 Introduction

The utilization of essential oils and extracts from aromatic plants and spices has been prevalent in various fields, such as food preservation, pharmaceuticals, alternative medicine, and natural therapies (Saini et al., 2021). Scientific investigation into these plants is imperative to understand the composition of their essential oils (EO) and their biological activities, which have been traditionally employed in medicinal practices to enhance healthcare quality (Mahleyuddin et al., 2021). This study focuses on *Coriandrum sativum* L. (*C. sativum*), a spice and medicinal plant that holds significance due to its essential oil content in leaves, stems, flowers, and fruits/seeds (Nouioura et al., 2023).

Coriander is an herbaceous annual plant belonging to the family Umbelliferae/Apiaceae, renowned for its aromatic properties. It has a rich history as a culinary herb, contributing aroma compounds and essential oils with bioactive components exhibiting antibacterial, antifungal, and antioxidant activities (Naeemasa et al., 2015). Coriander finds applications in food preparation, serving as a flavoring agent, and aiding in food preservation by preventing foodborne diseases and spoilage (Nejad Ebrahimi et al., 2010).

*C. sativum* provides two types of herbal raw materials: fruits and leaves. The main biologically active substance is the essential oil, with coriander fruits and seeds acting as an aromatic spice in dishes, also facilitating the digestion process. The yield and chemical composition of *C. sativum* essential oil undergo changes during ontogenesis, influencing the plant’s aroma (Jia et al., 2021).

Recent studies have continued to explore the properties and applications of coriander oil, building upon previous research. For example, a recent study by Silva et al., investigated the antimicrobial activity of coriander oil, particularly focusing on its major component, linalool. This study corroborated earlier findings regarding the ability of linalool to enhance membrane permeability in negatively charged membranes (Silva and Domingues, 2017).

Another study by Jabeen et al., examined the diuretic activity of coriander essential oil on Wistar rats of either sex (weighing 200–250 g). Their findings concluded that coriander exhibited a significant diuretic effect, which is considered advantageous for the treatment and management of uncomplicated hypertension (S. et al., 2014).

Known as “kuzbar” or “kuzbura” in Arabic, coriander is native to the Mediterranean and Middle Eastern regions (Msaada et al., 2017). Approved for food use by regulatory bodies, including the US Food and Drug Administration, the Flavor and Extract Manufacturers Association, and the Council of Europe, coriander serves as a spice, medicine, and raw material in the food, beverage, and pharmaceutical industries. In addition, its essential oil is esteemed for their therapeutic, medicinal, and industrial properties (Sriti et al., 2011). Due to its antimicrobial and antioxidant properties, CS-EO is considered a potential alternative natural preservative and nutraceutical ingredient (De Figueiredo et al., 2004).

The study aimed to explore the therapeutic potential of Moroccan coriander. Gas Chromatography-Mass Spectrometry (GC-MS) was employed to analyze the biochemical components of coriander fruits and seed essential oil. Selected *in vitro* and *in silico* assays were conducted to assess antioxidant activities, including DPPH, ABTS, BTBC, RP, and TAC, as well as antimicrobial activity against multi-resistant pathogenic microorganisms.

## 2 Materials and methods

### 2.1 Plant materiel and EO extractions

*C. sativum* L. fruits and seeds were harvested in the Sefrou region (33° 410 4500 N,4° 220 1800 W) in July 2022, according to the most recent WHO guidelines (WHO, 1998). Botanical authentication was conducted at the Scientific Faculty, Sidi Mohammed Ben Abdellah University in Fez, Morocco, under voucher specimen RAB76745. The extraction of CS-EO was carried out through hydro-distillation using a Clevenger-type apparatus. In brief, 100 g of dry seeds was placed in a 2 L flask with 1 L of distilled water and boiled for 3 h. The resulting oil was collected and stored at a temperature of 4°C until the subsequent assays. At least three separate extractions were performed, and the mean yield and standard deviation were calculated.

### 2.2 Chemical characterization of essential oil by GC/MS

The chemical composition of the CS-EO was determined using gas chromatography (TRACE GC-ULTRA, S/N 20062969, Thermo-Fisher Scientific, Waltham, MA, USA) coupled with mass spectrometry (Quadrapole, PolarisQ, S/N 210729, Thermo-Fisher Scientific, Waltham, MA, USA). Analysis was conducted using a capillary column (HP-5MS) with a length of 50 m, an internal diameter of 0.32 mm, and a film thickness of 1.25 μm. The temperature program ranged from 40°C to 280°C with an increment of 5°C per minute. The injector and detector (Polaris Q) were maintained at temperatures of 250°C and 200°C, respectively. Ionization was performed in electron-impact mode (EI) at 70 eV.

Helium was used as the carrier gas with a flow rate of 1 mL/min and a split ratio of 1:40. For analysis, 1 μL of the EO was injected. The identification of EO components was accomplished by comparing their retention times to those stored in the NIST-MS Search Version 2.0 library, allowing for the calculation of component percentages.

### 2.3 Antioxidant activity of CS-EO

Five methods to unveil the antioxidant activity of CS-EO were used: 2,2-Diphenyl-1-picrylhydrazyl radical (DPPH^•^), 2,2′-azino-bis (3ethylbenzothiazoline-6-sulfonic acid) radical cation-based (ABTS^•+^), Linoleic acid/b-carotene bleaching assay (BCBT), reducing antioxidant power (RP) assays, and total antioxidant activity (TAC), (Ferreira-Santos et al., 2019). All assays were determined spectrophotometrically using a UV/Vis spectrophotometer.

For the DPPH assay, a calibration curve was prepared using BHT (Butylated hydroxytoluene) with a concentration range of 100–5 mmol/L (*R* ^2^ = 0.986) and 434–11 mmol/L (*R* ^2^ = 0.992) for the ABTS assay, with a Trolox control. For the both RP and TAC assay, the ascorbic acid was standard with reference (1,000–100 μM, *R* ^2^ = 0.996). RP values are expressed as μg/g of Asc ac equivalent per g of CS-EO (µg AAE/g), and (mg AAE/g Dw) for the TAC test.

Equation 1 demonstrates the calculation of the radical scavenging activity for the DPPH and ABTS methods in % inhibition. Additionally, the terms of the residual color inhibition are determined by Eq. 2.

$$\text{Inhibition }\left(\%\right)=\frac{\text{Abs}\;\text{control}-\text{Abs}\;\text{sample}}{\text{Abs}\;\text{control}}\times 100$$ (1)

$$\text{Inhibition }\left(\%\right)=\frac{{\text{Abs}\;}_{\left(t=100\;\min\right)}\;}{{\text{Abs}\;}_{\left(t=0\right)}}\times 100$$ (2)

The results were expressed concentrations required to inhibit 50% (IC_50_) of DPPH^•^, ABTS^•+^ and BCBT radicals (µg/mL).

### 2.4 Antimicrobial activity

#### 2.4.1 Microbial strains

A total of five microbial strains comprised two Gram-negative bacteria; *Escherichia coli* ATCC 25922, and *Salmonella enterica serotype Typhi* and two Gram-positive, namely, *Staphylococcus aureus* ATCC 29213 and *Bacillus subtilis* ATCC 6633, and one fungal strain, *Candida albicans* (clinical isolates) were used in these exploratory investigations. All the microbial strains used in the current experiment were procured from the Laboratory of Microbiology situated at the Faculty of Sciences, Fez, Morocco.

#### 2.4.2 Disc-diffusion assay

The antimicrobial effectiveness of CS-EO was evaluated using the agar disc-diffusion method, with slight modifications as delineated elsewhere (Al-Mijalli et al., 2022) with some reforms. In short, bacterial culture suspensions were introduced onto Luria-Bertani (LB agar) medium for bacteria and onto Yeast Extract-Peptone-Dextrose Agar (YPD agar) for *Candida* species. Prior to their placement on the agar plates, sterile paper discs with a 6 mm diameter were impregnated with 10 μL of pure CS-EO. The positive control was established using discs saturated with Fusidic acid (10 μg/disc). Microbial cultures were incubated for a 24-h period within a temperature range of 30°C–35°C for bacteria. Regarding the antifungal assessment, the incubation was conducted at 25°C for 48 h. Following the incubation period, the diameters of the inhibitory zones were meticulously measured in millimeters, and the results were reported as the mean value ±standard error of the mean based on three independent experimental repetitions.

#### 2.4.3 Minimum inhibitory concentration assay (MIC)

The MIC of CS-EO was determined following a previously documented method (El Hachlafi, Benkhaira, Zouine, et al., 2023), with minor adjustments. Serial two-fold dilutions of the EO were prepared, spanning concentrations from 8.0% to 0.0625% (v/v) in Mueller–Hinton broth, supplemented with 5% dimethyl sulfoxide (DMSO). It is worth noting that prior studies have established that DMSO concentrations up to 7.8% do not exert a significant impact on the viability of microbial cells (Al-Bakri and Afifi, 2007), and our preliminary experiments confirmed that 5.0% DMSO does not affect microbial growth. Subsequently, 5 µL of a calibrated bacterial suspension was added to each well containing 100 μL of the serially diluted CS-EO, and 95 μL of sterile LB broth was introduced into all the wells. Negative controls, which consisted of all components without the bacterial suspension, were included, along with positive controls, (Fusidic acid). Following the designated incubation period, 20 mL of 2 mg/ML of p-iodonitrotetrazolium chloride was introduced into all micro-tubes. The plates were subjected to an additional 30-min incubation period, during which the presence of microorganism growth was signified by the appearance of a purple-red color, a consequence of the reduction of INT into formazan. The MIC was determined as the lowest concentration of the extract capable of inhibiting microbial growth following 24 h (bacteria) or 48 h (*Candida* species) of exposure to the CS-EO.

#### 2.4.4 MBC and MFC assessment

The minimum bactericidal concentration (MBC) and minimum fungicidal concentration (MFC) were evaluated by sub-culturing the test dilutions onto unseeded plates LB agar medium. Subsequently, these plates were further incubated for a period of 24 h for bacteria) or 48 h for *Candida* species). The highest dilution at which no individual bacterial colony was observed on the plates was designated as the MBC or MFC. Additionally, the ratios of MBC/MIC and MFC/MIC were also calculated (El Hachlafi, Benkhaira, Al-Mijalli, et al., 2023).

### 2.5 Drug-likeness and molecular docking simulations of CS-EO

In light of the experimental results that were achieved, and after discovering the chemical compounds contained in the *C. sativum* L., essential oil using GC/MS, technology, we examined its components based on *in silico* techniques, including the predictions of physical, chemical, and pharmacokinetic properties, which would make it an important source of effective treatments against antioxidant and antibacterial activities for several bacteria’s, more than molecular docking simulations which were carried out to discover the inhibition mechanisms for the major compounds of the examined essential oil towards antibacterial, antifungal, and antioxidant proteins.

The physicochemical features of extracted chemical compounds from this essential oil were initially predicted based on Lipinski’s five rule (Lipinski et al., 2001), to verify their similarities to small Candidate drugs. Subsequently, the pharmacokinetic properties of ADMET were equally tested for each chemical compound, using Swiss ADMET and pkCSM online servers (El Fadili et al., 2023). After that, the predictive model of Egan’s BOILED-Egg was applied to identify the potent central nervous system (CNS) agents, with the highest probability of crossing the blood-brain barrier (Daina et al., 2017). Finally, the oral bioavailability was also examined for the examined molecules using bioavailability radars, taking into account six physicochemical characteristics ideal for oral bioavailability, namely, lipophilicity, size, solubility, flexibility, saturation, and polarity (Kandsi et al., 2022). Thereafter, the major compounds of *C. sativum* L., essential oil was docked to 3D crystal structures of Water-forming NAD(P)H oxidase from *Lactobacillus sanfranciscensis*, DNA gyrase B protein from *E. coli*, and sterol 14-alpha demethylase (CYP51) from *C. albicans*, which were extracted from protein data bank (PDB) by codes of 2CDU, 6F86, and 5TZ, respectively. The targeted proteins were prepared using AutoDock 4.2 software (Norgan et al., 2011), removing all co-crystallized ligands bound to each responsible protein, adding the charges of gasteiger, and deleting the suspended water (H_2_O) molecules. Finally, three and two-dimensional interactions of the strongest (ligand-protein) complexes were visualized using Discovery Studio 2021 software (Systems, 2020).

## 3 Results and discussion

### 3.1 Phytochemical profile of *C. sativum* essential oil

The coriander oil obtained by hydrodistillation from dried fruits and seeds showed a green-yellow color and a strong characteristic odor. The yields of oils were ranges from 0.1% to 0.4%.

**FIGURE 1:**
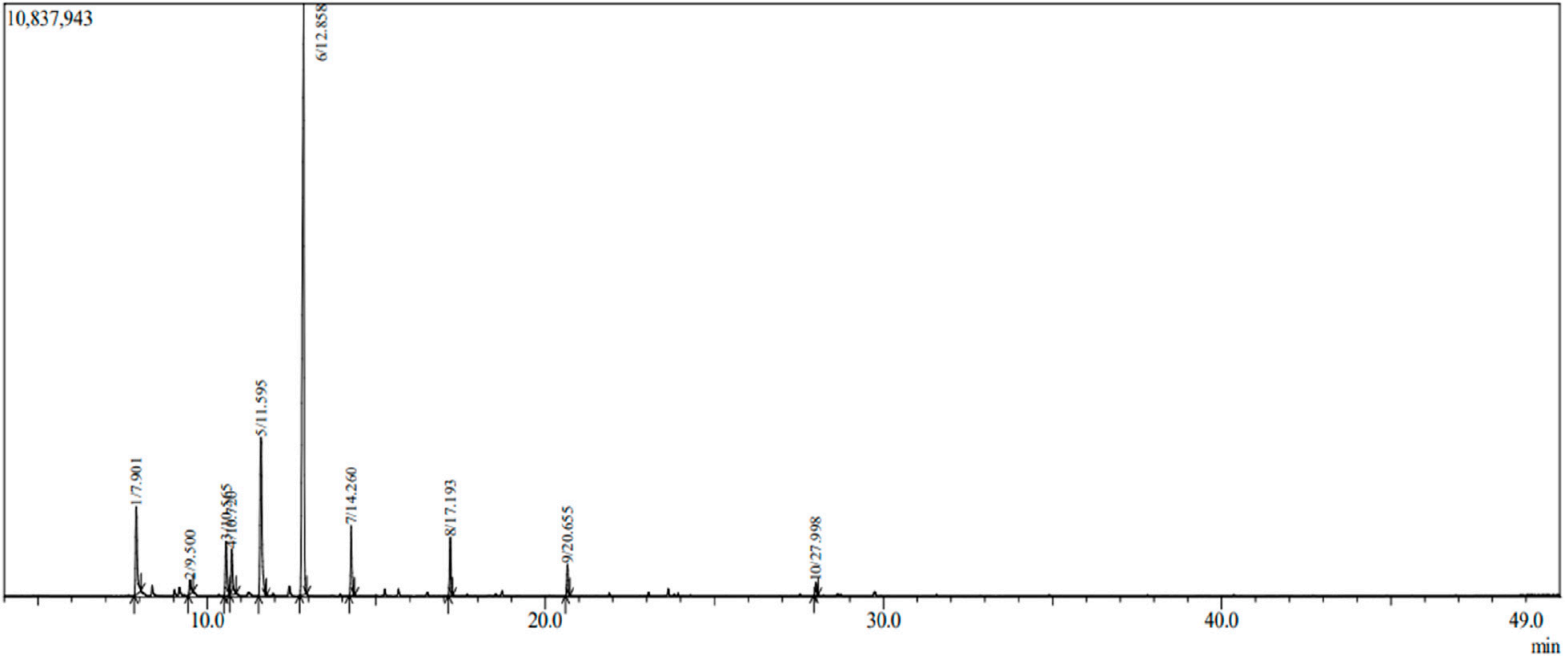
GC-MS chromatogram of essential oil extracted from *C. sativum* seeds.

Table 1 presents the composition of the essential oil as analyzed by GC-MS and identified by the Wiley Library and Kòvats index. Thirteen compounds were identified in CS-EO, with the major component being linalool (59.04%), followed by γ-terpinene (13.02%) and α-pinene (6.83%). Additionally, camphor and o-cymene constituted 5.43% and 4.30%, respectively, adding up to 100% of the total EO composition (Figure 1). In a study on Indian coriander fruits, Viuda-Martos et al., reported Geranyl acetate (46.27%) as the major component, followed by linalool (10.96%) (Viuda-Martos et al., 2011). Linalool and geranyl acetate were also found in Indian coriander seeds (75.30% and 8.12%, respectively) (Palmieri et al., 2020). Khani & Rahdari, in Iranian coriander seeds, also identified linalool (57.57%) as a major component of the EO (Khani and Rahdari, 2012).

**TABLE 1:** Principal constituents of organic coriander essential oils from Morocco.

No	RT (min)	Molecular formula	Compounds	Kováts indexes		Peak area%
				RIc	RI lit	
1	7.90	C_10_H_I6_	α -Pinene	933	948	6.83
2	9.50	C_10_H_I6_	β-Myrcene	958	980	1.12
3	10.56	C_10_H_I4_	o-Cymene	1,042	1,025	4.30
4	10.72	C_10_H_16_	D-Limonene	1,018	1,036	3.83
5	11.59	C_11_H_18_	γ-Terpinene	1,059	1,068	13.02
6	12.85	C_10_H_18_O	Linalool	1,082	1,100	59.04
7	14.26	C_10_H_8_O	camphor	1,121	1,139	5.43
8	17.19	C_10_H_8_O	Geraniol	1,228	1,235	3.66
9	20.65	C_12_H_20_O_2_	Geranyl acetate	1,352	1,383	1.84
10	27.99	C_12_H_14_O_2_	Butylphthalide	1,526	1,527	0.94
	Chemical classes					
	Oxygenated monoterpenes					69.97
	Monoterpene hydrocarbons					29.1
	Others					0.93
	Total (%)					100

Sriti et al., studied the chemical composition of CS-EOs from Tunisia and reported the main components of Coriander EO as 79.22% linalool, followed by 6.26% terpinene (Sriti et al., 2011). Masmada et al., reported that geranyl acetate (46.27%) was the main component, followed by fenchone, α-pinene, methyl-chavicol, α-phellandrene, and d-limonene (Msaada et al., 2017).

Linalool (2,6-dimethyl-2,7-octadian-6-ol) is an aromatic monoterpene alcohol widely employed in perfumes, cosmetics, household cleaners, and food additives. It exhibits the capacity to cause oxidative stress-induced apoptosis in cancer cells while simultaneously shielding healthy cells. By rupturing cell membranes, linalool demonstrates antimicrobial properties. Its protective impact on the liver, kidneys, and lungs is attributed to its anti-inflammatory activity. Linalool is a possible adjuvant for antibiotics or anticancer medications because of its low toxicity and protective qualities. Thus, linalool has a great deal of promise as a safe and natural alternative medicine.

Various factors, encompassing both endogenous and exogenous elements, exert influence on the chemical composition of essential oils. Endogenous factors are linked to the anatomical and physiological properties of plants, as well as the biosynthesis pathways of volatiles (Al-Snafi, 2016). These pathways may exhibit variation based on the plant’s tissue, season, and DNA adaptation. Over an extended period of time, exogenous factors can affect specific genes responsible for volatile formation (Barra, 2009). In the context of the same plant species, these variables give rise to ecotypes or chemotypes (Demir and Korukluoglu, 2020).

### 3.2 Antioxidant activity of CS-EO

Five recommended *in vitro* assays, including DPPH, ABTS, BCBT, RP, and TAC, were used to measure the antioxidant activity of the investigated of CS-EO.

Free radical scavenging activity, assessed through colorimetric methods such as DPPH and ABTS, is commonly employed to evaluate the antioxidant properties of different substances found in plants, foods, beverages, or natural extracts (De Menezes et al., 2021). These tests operate by reducing chemical radicals when antioxidants donate hydrogen. These methods are considered valid, straightforward, fast, precise, dependable, and cost-effective for evaluating natural antioxidants’ free radical scavenging activity, as the radicals used in these tests are stable and readily available. The DPPH assay measures the ability of the examined extracts to scavenge free radicals by converting DPPH• (radical) to the non-radical DPPH-H form in the presence of hydrogen-donating antioxidants. The ABTS test is fundamentally rooted in the chemical interaction between particular antioxidant compounds and the ABTS radical cation (ABTS^•+^). This interaction results in the suppression of hydrogen atoms by the nitrogen atom of ABTS^•+^, subsequently causing the mixture to lose its coloration (Shekhar and Anju, 2014).

The method, commonly referred to as “β-carotene bleaching test”, involves the oxidation of both β-carotene and linoleic acid. This technique assesses the ability of antioxidant compounds present in plant extracts to neutralize the linoleic acid peroxide radical, which, in turn, prevents the oxidation of β-carotene during the emulsion phase. In the absence of antioxidants, β-carotene rapidly undergoes discoloration, attributed to the attack by the free linoleic acid radical, leading to the breakage of the molecule’s double bonds (Miguel, 2010).

The other process employed in this study, known as RP, is a colorimetric assay that relies on the ability of antioxidant molecules to reduce ferric ions (Fe^3+^ to Fe^2+^) by acting as electron-donating antioxidants (Thaipong et al., 2006). The ammonium phosphomolybdate assay used to determine the total antioxidant capacity has been widely used on biological samples to evaluate extracellular non-enzymatic antioxidants (Wang et al., 1996).

A more comprehensive approach involves employing a spectrum of assessments for individual antioxidants and indicators of oxidative damage, with total antioxidant capacity (TAC) potentially serving as one of these evaluations. A diminished TAC level might indicate the presence of oxidative stress or an elevated vulnerability to oxidative harm (Young, 2001).

As depicted in Table 2, the IC_50_ value of DPPH radical scavenging activity of coriander sativum essential oil was 1.52 ± 0.14 mg/mL, whereas standard BHT showed much higher efficiency (0.024 ± 0.00 mg/mL). An earlier study stated that CS-EO showed a higher DPPH IC_50_ value of 38.83 ± 0.76 μg/mL. Furthermore, it was reported by Sriti et al., that the essential oils of two coriander fruits fluctuated between 60000 and 61000 μg/mL (Sriti et al., 2011).

**TABLE 2:** Antioxidant activities of CS-EO.

	CS- EO	BHT	TEAC	Asc Ac
DPPH IC_50_(mg/mL)	1.52 ± 0.14	0.024 ± 0.00	-	-
ABTS IC_50_(mg/mL)	0.51 ± 0.04	-	0.02 ± 0.00	-
BCBT IC_50_(mg/mL)	9.02 ± 0.01	-	-	0.032 ± 0.00
RP EC_50_(µg/mL)	6.13 ± 0.00	-	-	10.24 ± 0.07
TAC (mg/mL)	213.44 ± 0.45	-	-	-

ABTS: 2,2′-Azinobis-(3-Ethylbenzthiazolin-6-Sulfonic Acid, Asc Ac: Ascorbic acid, BCBT: β-carotene bleaching test, BHT: 2,6-di-tert-butyl-4-methylphenol, DPPH: 2,2-diphenyl 1-picrylhydrazyl, RP: reducing power, TAC: total antioxidant capacity, TEAC: trolox equivalent antioxidant capacity.

The ABTS test revealed even higher antiradical capacities, with an IC_50_ value of 0.51 ± 0.04 mg/mL, which exhibited the strongest antioxidant activity but was about two times weaker than the reference drug. These values, although lower than those reported by Derouich et al., where IC_50_ values were 102.95 μmol TE/g (Derouich et al., 2020), Superoxide anion and β-carotene assays were more successful than DPPH and ABTS assays, following a similar pattern and yielding a result of 9.02 ± 0.01 mg/mL. With an IC_50_ = 25.70 ± 1.02 μg/mL (Hajlaoui et al., 2021), our results were determined strongly when compared with Hajlaoui et al.'s study. On the other hand, Sriti et al. reported that the IC_50_ of CS-EO was 56000 ± 2.65 μg/mL (Sriti et al., 2011).

Comparing the RP assay results to the ascorbic acid standard (10.24 ± 0.07 μg/mL), Table 2 shows a comparatively high EC_50_ value of 6.13 ± 0.00 μg/mL. These findings surpass those reported by Hajlaoui et al., for Tunisian coriander, where the RP value was 24.00 ± 1.53 μg mL^−1^/EDTA (Hajlaoui et al., 2021).

Our essential oil has an impressive total antioxidant capacity value of 213.44 ± 0.45 mg AAE/mL of EO, according to Table 2’s results, when compared to standards of 257.26 ± 0.21 mg EAA/g for quercetin and 269.2 ± 0.10 mg EAA/g for BHT.

The elevated terpene content of CS-EO is responsible for its high antioxidant potential. A pre-aromatic monoterpene hydrocarbon with non-phenolic properties, γ-terpinene has been shown to have antioxidant properties and to be able to prevent lipid peroxidation (Demir and Korukluoglu, 2020). Supplementary or alternative methods to improve the oxidative stability and shelf life of edible lipids, similar to adding vitamin E, could involve incorporating γ-terpinene-rich EOs (Al-Snafi, 2016). Linalool, which is abundant in *C. sativum*, has a conjugated double bond and significant antioxidant activity, which help to explain its strong capacity to scavenge free radicals (Jia et al., 2021). It has also been demonstrated that provides defense against lipid peroxidation (Silva and Domingues, 2017). As a synergistic rather than a lone antioxidant, the alcohol linalool, which is predominant in *C. sativum* EO, is essential to the EO’s activity (Priya et al., 2022). In complex natural mixtures, the monoterpene p-cymene is thought to possess strong antioxidant activity (Palmieri et al., 2020). The strong antioxidant effect of the EO mixture in our study is partially explained by the synergistic effects that p-cymene produces when it interacts with other monoterpenes, demonstrating its potent antioxidant power.

In order to verify this activity, we ran additional antimicrobial tests on CS-EO due to its notable antioxidant capacity.

### 3.3 Antimicrobial activity of CS-EO

The disc diffusion method was employed to evaluate the inhibitory effects of Moroccan organic *C. sativum* essential oils (EOs) against *B*. *subtilis* ATCC 6633, *S*. *enterica serotype* Typhi, *E*. *coli* ATCC 27853, and *S*. *aureus* ATCC 29213, and *C*. *albicans*. Table 3 outlines the inhibitory halos and antibacterial/antifungal properties of the CS-tested oil. EO activities were categorized as low (<10 mm), moderate (>10–15 mm), and high (>15 mm) based on the inhibition zone diameter (Silva and Domingues, 2017). The CS-Eo exhibited the highest antibacterial activity against *S*. *enterica* serotype Typhi (28.83 ± 2.02 mm), followed by *B*. *subtilis* (17.06 ± 0.90 mm), and *E*. *coli* (10.26 ± 0.64 mm), while moderate activity was observed against *S*. *aureus* (9.40 ± 0.52 mm).

**TABLE 3:** Antibacterial effect of *C. sativum* essential oils using the Disc diffusion method.

Bacteria^a^	Diameter of inhibition zone (mm ± SD)^b^	
	CS-EO	Fusidic acid (10 μg/disc)
*Bacillus subtilis* ATCC 6633	17.06 ± 0.90^b^	14.63 ± 2.22^b^
*Salmonella enterica* serotype Typhi	28.83 ± 2.02^a^	10.63 ± 1.65^b^
*Escherichia coli* ATCC 27853	10.26 ± 0.64^b^	15.70 ± 0.65^a^
*Staphylococcus aureus* ATCC 29213	9.40 ± 0.52^c^	14.06 ± 0.61^b^
Fungic^a^	CS-EO	Fluconazole
*Candida albicans*	12.43 ± 0.57	12 ± 1.05

Fusidic acid was used as positive control. Results are expressed as means ± SD, of three independent measurements.

^a^ Final microbial density was around 10^6^ CFU/mL for bacteria and10^4^ CFU/mL for *Candida* species.

^b^ Diameter of inhibition zone including disc diameter of 6 mm (10 μL of oil/disc). Data with the same letter in the same test present non-significant difference by Tukey’s multiple range test (ANOVA, *p* < 0.05).

In comparison to reference antibiotics, Gram-positive bacteria were more susceptible, and these findings were statistically significant (ANOVA, *p* < 0.05). In terms of antifungal potential, disc diffusion demonstrated moderate activity against *C*. *albicans* (12.43 ± 0.57 mm); the outcomes were noteworthy and similar to the antifungal medication used as a reference (Table 3). The broth microdilution method determined MIC, MBC, and MFC values (Table 4). The lowest MIC and MBC values were recorded with Gram-positive bacteria (*B. subtilis, S. enterica, S. aureus*), ranging from 0.0612% to 0.25% v/v for MIC and 0.125%–0.5% v/v for MBC. For Gram-negative bacteria (*E. coli*), MIC and MBC values were 1.0% v/v. These results supported the disc-diffusion method findings (Table 2). For the *C*. *albicans* fungal strain, MIC = 0.5% v/v, and MFC = 1.0%, confirming the notable antifungal efficacy of CS-EO. MBC/MIC and MFC/MIC ratios indicated that CS-EO has a bactericidal and fungicidal mechanism.

**TABLE 4:** Antibacterial effect of CS-EO using broth microdilution method.

Bacteria	CS-EO			Fusidic acid (µg/mL)		
	MIC	MBC	MBC/MIC	MIC	MBC	MBC/MIC
*B. subtilis* ATCC 6633	0.125	0.125	1.0	32	32	1.0
*S. enterica* serotype Typhi	0.0612	0.125	2.0	64	32	2.0
*E. coli* ATCC 27853	1.0	1.0	1.0	356	356	1.0
*S. aureus* ATCC 29213	0.25	0.5	2.0	64	64	1.0

Fungi	CS-EO			Fluconazole (µg/mL)		
	MIC	MFC	MFC/MIC	MIC	MFC	MFC/MIC
*Candida albicans*	0.5	1.0	2.0	32	64	2.0

MIC: Minimum inhibitory concentration in % (v/v), MBC: Minimum Bactericidal concentration in % (v/v). Fusidic acid and fluconazole were used as standard drugs.

This conclusion is based on the observation that antimicrobial agents fall into the bactericidal or fungicidal category if the ratio of MBC/MIC to MFC/MIC is 4.0 or lower. According to this ratio, it should be possible to obtain tested agent concentrations that are capable of eliminating 99.9% of the treated microorganisms. Ratios higher than 4.0 could suggest difficulties in administering dosages high enough to kill microorganisms at the same level, designating the substance as bacteriostatic (El Hachlafi et al., 2023).

It has been demonstrated that the higher resistance of Gram-negative bacteria relative to Gram-positive bacteria may be attributed to the thick layer of peptidoglycan on their cell walls, which impedes the passage of antimicrobial agents and provides their cells with rigidity (Hajlaoui et al., 2021). Terpenes constitute 75% of antibacterial medications, as previously mentioned (Khani and Rahdari, 2012). Depending on the type of essential oil and the microorganism in question, EOs can exhibit varying antimicrobial effects. This study particularly noted the robust antimicrobial activity of CS-EO, primarily due to its abundance of monoterpenes. Among them, linalool’s ability to inhibit the growth of fungi and bacteria has been well documented (Prachayasittikul et al., 2018). An antimicrobial study conducted on resistant *Klebsiella pneumoniae* demonstrated the efficacy of linalool, extracted from lavender essential oil, through membrane disruption (Laribi et al., 2015). Additionally, it is known that γ-terpinene exhibits potent inhibitory activity against *S*. *typhimurium* and *E. coli* (S. et al., 2014). Earlier research by Giweli et al., demonstrated the potent antibacterial activity of γ-terpinene, found in high concentrations in *C. sativum* (Giweli et al., 2013). In coriander oil, linalool, its major component, is primarily found in the form of its S (+) enantiomer (Silva and Domingues, 2017), known to induce increased permeability only in negatively charged membranes (Dėnė et al., 2022). Moreover, alcohols have been associated with a decrease in the thickness of the cell wall in Gram-positive bacteria, leading to increased resistance of bacteria to compounds such as solvents and dyes (De Figueiredo et al., 2004). In conclusion, the S enantiomer of linalool could cause disruption of the outer membrane of negatively charged Gram-negative bacteria, while its alcohol properties could lead to increased resistance of Gram-positive bacteria due to thickening of the bacterial cell wall as described above.

Both Gram-positive and Gram-negative bacteria are susceptible to the bactericidal effects of coriander oil, leading to membrane permeabilization and subsequent loss of all cellular functions, including efflux activity, metabolic activity, and membrane polarization, within 30 min of incubation (Silva and Domingues, 2017).

Although the overall mechanism of action of coriander oil remained the same, the results of Silva et al.'s analysis of differential bacterial susceptibility suggested that coriander oil may have distinct effects on Gram-positive and Gram-negative bacteria. This is attributed to the differences in cellular envelopes and oil compositions of the two bacterial species (Silva et al., 2011). According to the authors, the alcohols in the oil caused thickening of the cell wall in Gram-positive bacteria at low oil concentrations (Foudah et al., 2021).

Regarding the antifungal mechanism of action, even at concentrations below the MIC value, coriander oil prevents yeasts like *C*. *albicans* from forming germ tubes (Silva et al., 2011). CS-EO appears to target the fungal membrane, similar to what Silva and co-authors verified for bacteria. This is because, within 30 min of contact, all fungal cells exhibit membrane permeabilization, leading to the leakage of large intracellular components like DNA (Silva, Ferreira, Duarte, et al., 2011).

### 3.4 Drug likness, ADME-Toxicity predictions

*In silico* predictions of physicochemical properties, confirm that all extracted compounds from *C. sativum* L., essential oil satisfy Lipinski rules of five, as the molecular wights (MW) are inferior or equal to 500 g/mol, molar Refractivity indexes (MR) are comprised in the closed interval of [40, 130], the numbers of Hydrogen bond donors (HBD) exceed not five threshold, Lipophilicities expressed by (Log P _Octanol/Water_) are less than 5, and finally the numbers of Hydrogen bond acceptors (HBA) are all inferior or equal to 10, as resulted in Table 5. Therefore, all examined compounds were predicted as small molecules having significant resemblances to *candidate*-drugs (Assaggaf et al., 2023; Benkhaira et al., 2023; El fadili et al., 2023).

**TABLE 5:** Prediction of Lipinski physico-chemical features for ten molecules extracted from *C. sativum* L., essential oil.

Compounds number	Physico-chemical properties					Lipinski’s five rules
	MW	MR index	Log P	HBA	HBD	(No/Yes)
Rule	≤500(g/mol)	130≥ MR index ≥40	<5	≤10	<5	
C1	136.23	45.22	2.63	0	0	Yes
C2	136.23	48.76	2.89	0	0	Yes
C3	134.22	45.99	2.43	0	0	Yes
C4	136.23	47.12	2.72	0	0	Yes
C5	136.23	47.12	2.73	0	0	Yes
C6	154.25	50.44	2.70	1	1	Yes
C7	152.23	45.64	2.12	1	0	Yes
C8	154.25	50.40	2.52	1	1	Yes
C9	196.29	60.13	3.27	2	0	Yes
C10	190.24	54.99	2.53	2	0	Yes

C1: α -pinene, C2: β-myrcene, C3: o-cymene, C4: D-limonene, C5: γ-Terpinene, C6: linalool, C7: camphor, C8: geraniol, C9: geranyl acetate, C10: butylphthalide.

**FIGURE 2:**
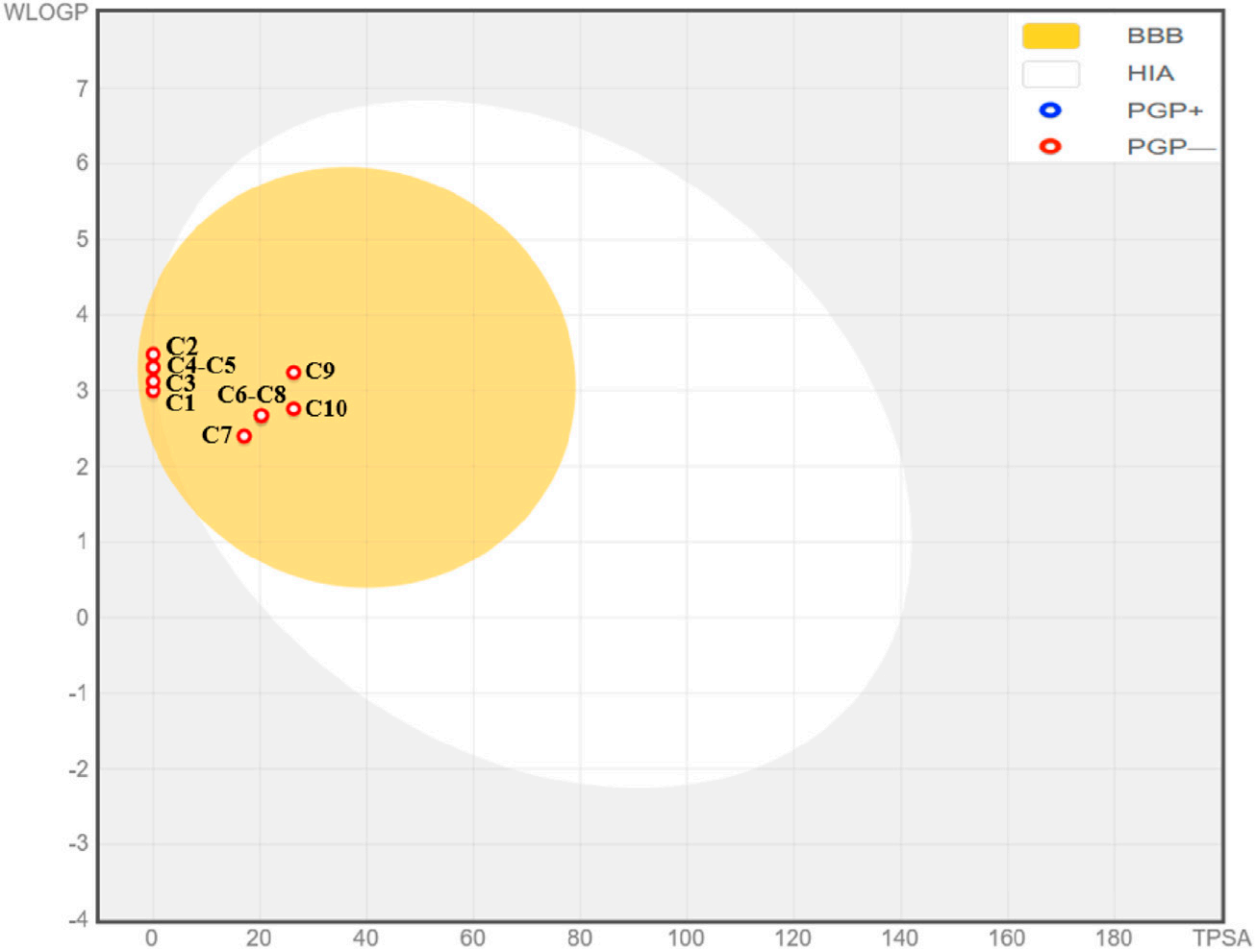
Predictive model of Egan’s boiled Egg.

**FIGURE 3:**
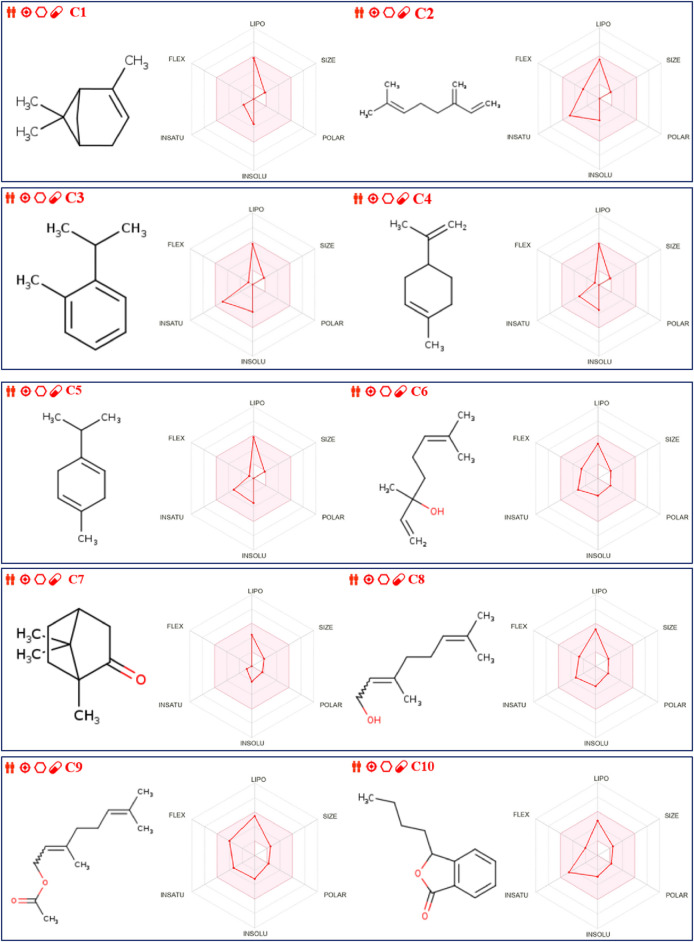
Bioavailability radars for ten extracted molecules from *C. sativum* L., essential oil.

Additionally, the analysis of ADME and Toxicity profile confirms that all extracted molecules were predicted with excellent human intestinal absorptions exceeding 93%, so they are predicted to be well absorbed by the human body. Next, all ten molecules were predicted to be well permeable to central nervous system and blood-brain barrier as their permeabilities are included in the ranges of [0, 1] Log PS and [-1, −3] Log BB, respectively. No inhibitory effect of extracted compounds on the cytochromes, just for C3 and C10 chemical compounds, which were predicted as potent inhibitors of 1A2 cytochrome. Moreover, the Ames toxicity test confirms that all extracted molecules were predicted with safety and security on human organism, without any undesirable effects of hepatotoxicity, except for C10 compound. However, the majority of these compounds were predicted with a positive skin sensitization effect on human body, as resulted in Table 6. The predictive model of Egan, confirm that all extracted compounds from *C. sativum* L., essential oil are part of yellow area of Egan’s egg, so they were predicted to traverse the blood-brain barrier with highest probability. Therefore, they are considered as potent inhibitors of central nervous system, as presented in Figure 2. Finally, the biovailability radars reveled that the essential oil under investigation, was predicted with excellent level of oral biovailability, because their chemical components (C1 to C10) were all located in the pink part of radars, as the appropriate area of biovailabilities based on six physicochemical properties of lipophilicity, size, solubility, flexibility, saturation, and polarity (Jeddi et al., 2023), as pictured in Figure 3.

**TABLE 6:** Prediction of ADME-Tox features for ten molecules extracted from *C. sativum* L., essential oil.

Compounds number	A	D		M							E	T		
	Human intestinal absorption	Blood-brain barrier permeability	Central nervous system permeability	Substrate		Inhibitor					Total clearance	AMES test of toxicity	Hepatotoxicity	Skin sensitization
				Cytochromes										
				2D-6	3A-4	1A-2	2C-19	2C-9	2D-6	3A-4				
	(% absorbed)	(Log BB)	(Log PS)	(No/Yes)							Numeric (Log mL/min/kg)	(No/Yes)		
C1	96.041	0.791	−2.201	No	No	No	No	No	No	No	0.043	No	No	No
C2	95.155	0.792	−1.912	No	No	No	No	No	No	No	0.438	No	No	No
C3	95.299	0.492	−1.338	No	No	Yes	No	No	No	No	0.259	No	No	**Yes**
C4	95.898	0.725	−2.37	No	No	No	No	No	No	No	0.213	No	No	**Yes**
C5	95.716	0.74	−2.023	No	No	No	No	No	No	No	0.217	No	No	No
C6	93.649	0.608	−2.28	No	No	No	No	No	No	No	0.446	No	No	**Yes**
C7	96.883	0.625	−2.178	No	No	No	No	No	No	No	0.109	No	No	**Yes**
C8	93.467	0.621	−2.179	No	No	No	No	No	No	No	0.437	No	No	**Yes**
C9	95.579	0.581	−2.219	No	No	No	No	No	No	No	0.587	No	No	**Yes**
C10	95.682	0.238	−1.918	No	No	Yes	No	No	No	No	0.808	No	**Yes**	**Yes**

A: absorption; D: distribution; M: metabolism; E: excretion; T: toxicity.

### 3.5 Molecular docking analysis

**FIGURE 4:**
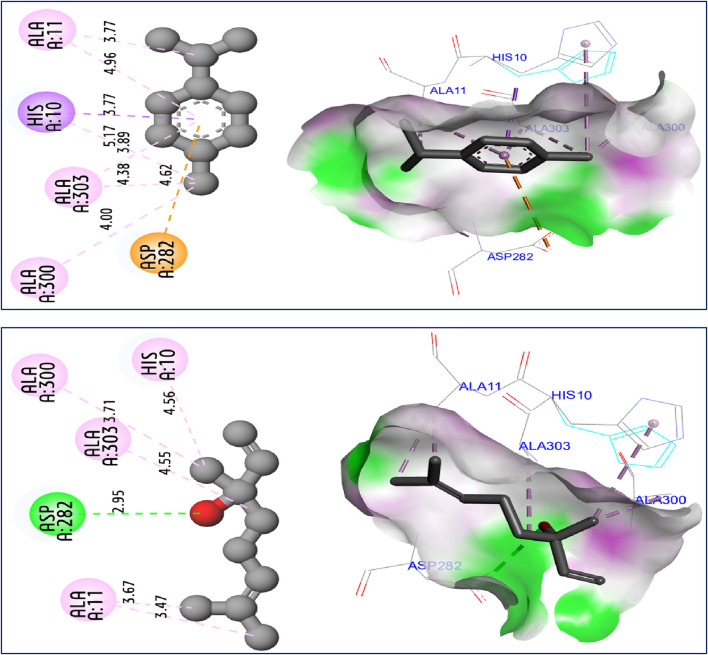
2D and 3D views of intermolecular interactions produced between C5, and C6 ligands towards 2CDU.pdb encoded protein, with binding energies of −5.94 and −5.22 kcal/mol, respectively.

**FIGURE 5:**
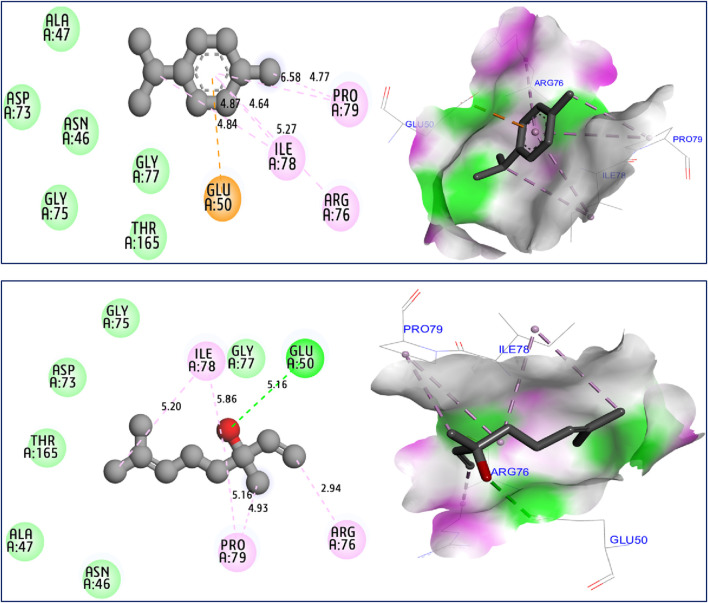
2D and 3D views of intermolecular interactions produced between C5, and C6 ligands towards 6F86.pdb encoded protein, with binding energies of −4.63 and −4.04 kcal/mol, respectively.

**FIGURE 6:**
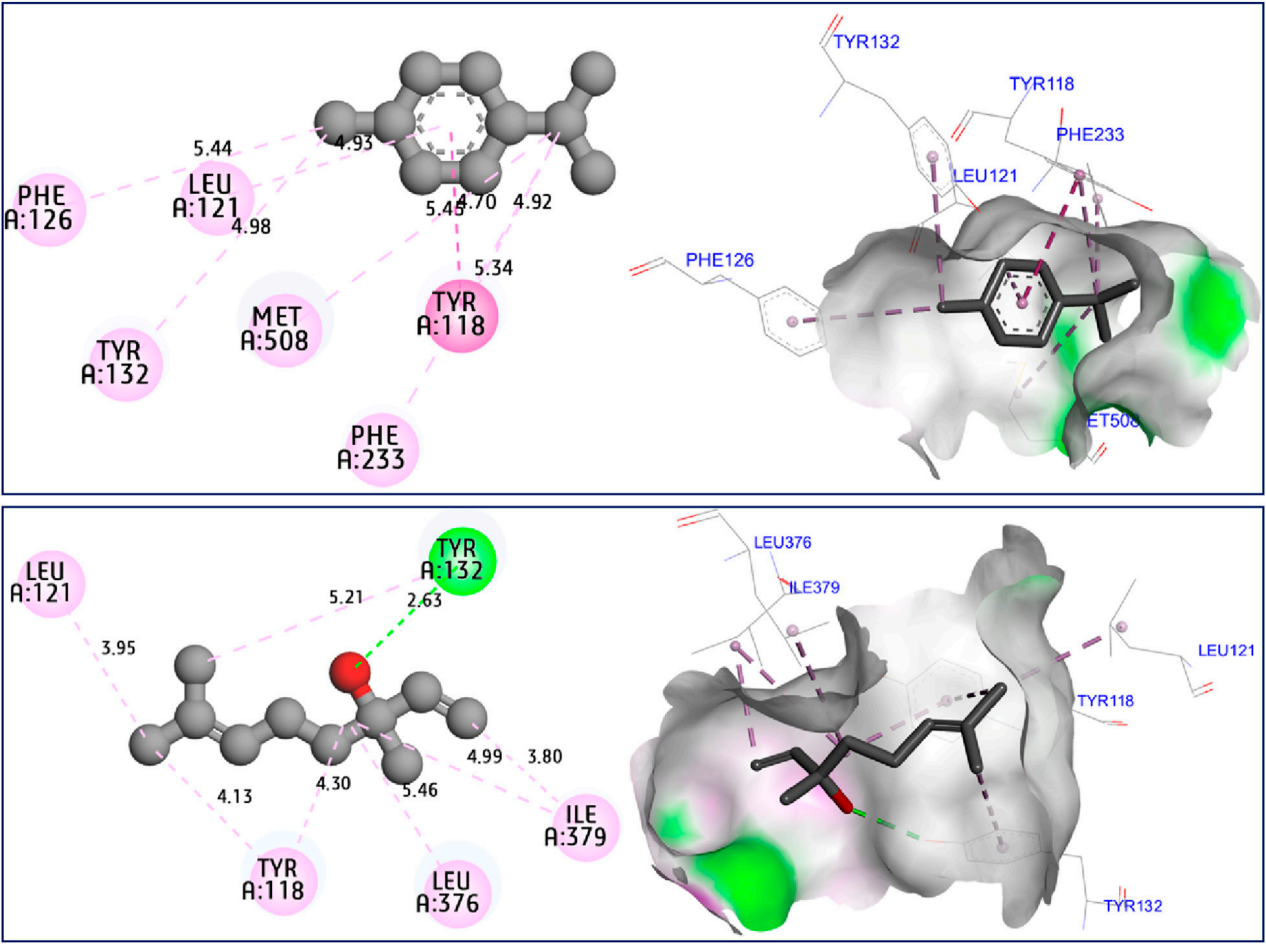
2D and 3D views of intermolecular interactions produced between C5, and C6 ligands towards 5TZ1.pdb encoded protein, with binding energies of −5.37 and −5.18 kcal/mol, respectively.

The results of molecular docking simulations clearly show from both 2D and 3D visualizations that the major compounds of *C. sativum* L., essential oil, namely, γ-terpinene and linalool, labeled C5 and C6 respectively, were complexed to NADPH oxidase protein (2CDU.pdb) (Jeddi et al., 2023; Nouioura et al., 2024), producing various intermolecular interactions with lowest binding energies of −5.94 and −5.22 kcal/mol, respectively. Among these interactions, common chemical bonds were created by the major compounds of the examined essential oil, as those detected towards Asp282, Ala11 and Ala300 active sites, more than Ala303 and His10 amino acid residues, as resulted in Figure 4. The main compounds were equally docked to DNA gyrase B protein from Escherichia coli, encoded as 6F86.pdb (Yuan and Hao, 2023; Nouioura et al., 2024), with binding energies of −4.63 and −4.04 kcal/mol respectively, revealing common interactions like as chemical bonds detected with Glu50, Ile78, Pro79, and Arg76 amino acids residues, as displayed in Figure 5. The same major compounds have been docked another time to sterol 14-alpha demethylase (CYP51) from *C*. *albicans* coded 5TZ1.pdb (Gandham et al., 2024; Nouioura et al., 2024), with binding energies of −5.37 and −5.18 kcal/mol respectively, sharing two common interactions as the chemical bonds detected towards Tyr118 and Tyr132 active sites, more than Leu121 amino acid residue, as resulted in Figure 6. Therefore, the inhibition mechanism of antioxidant activity of *C. sativum* L., essential oil is justified by the production of chemical bonds with Asp282, Ala11, Ala300, Ala303, and His10 AARs, while the antibacterial effect is explained by the detection of chemical bonds with Glu50, Ile78, Pro79, and Arg76 AARs. So, the antifungal activity of studied essential oil is justified by the formation of common bonds with Tyr118, Tyr132, and Leu121 AARs.

## 4 Conclusion

In the present work, we investigated the chemical constituents, antioxidant capacity, and antimicrobial qualities of the essential oil extracted from *C. sativum* L., in relation to nosocomial, antibiotic-resistant microbes. As demonstrated by the GC-MS investigation, these effects are likely related to various bioactive compounds identified in the volatile part of CS-EO. According to the ADMET simulation, the primary constituents of CS-EO exhibit advantageous pharmacokinetic characteristics. Additionally, considering CS-EO as a promising natural agent with applications across numerous industries, it warrants significant attention.

Indeed, this oil holds potential uses in the food industry, particularly as active packaging (films and coatings). The eco-friendly and biodegradable qualities of CS-EOs also make them suitable for application as biopesticides in the agricultural sector. Furthermore, they may serve as effective nano-delivery systems in the pharmaceutical and medical sectors, presenting significant biomedical applications. To confirm the pharmacological effects of this plant, additional *in vivo* and clinical research is highly recommended. Additionally, determining its toxicity is essential for confirming its safety.

## Data Availability

The raw data supporting the conclusion of this article will be made available by the authors, without undue reservation.
